# Bacillus coagulans in Combination with Chitooligosaccharides Regulates Gut Microbiota and Ameliorates the DSS-Induced Colitis in Mice

**DOI:** 10.1128/spectrum.00641-22

**Published:** 2022-07-28

**Authors:** Zhenzhen Liu, Ziyang Jiang, Zhenting Zhang, Tong Liu, Yurong Fan, Tao Liu, Nan Peng

**Affiliations:** a Antibiotics Research and Re-evaluation Key Laboratory of Sichuan Province, Sichuan Industrial Institute of Antibiotics, School of Pharmacy, Chengdu University, Chengdu, People’s Republic of China; b State Key Laboratory of Agricultural Microbiology, Hubei Hongshan Laboratory, College of Life Science and Technology, Huazhong Agricultural University, Wuhan, People’s Republic of China; Lerner Research Institute

**Keywords:** *Bacillus coagulans*, chitooligosaccharides, synbiotic, microbiota, short-chain fatty acids

## Abstract

Ulcerative colitis (UC) are chronic inflammatory disorders, which may be caused by intestinal barrier dysfunction, immune system disorders and intestinal microbiota dysbiosis. Synbiotic, the combination of probiotics and prebiotics, is thought to be a pragmatic approach in mitigating inflammation in UC. Bacillus coagulans has been recognized as a potential probiotic for treating intestinal diseases because of its favorable industrial and probiotic properties, including sporulation and lactic acid production. In this study, we evaluated the treatment effects of the *B. coagulans* FCYS01 spores with or without the chitooligosaccharides (COSs) on UC generated using dextran sulfate sodium (DSS) in mice. Supplementation of *B. coagulans* spores, prebiotic COSs or the synbiotic (the spores + COSs) had a significant positive effect on DSS-induced UC. The disease activity index and histological damage score were significantly reduced after these supplementations. Compared to DSS group, these supplementations also significantly modulated the cytokines IL-4, IL-6, IL-8, IL-10, and C-reactive protein (CRP) levels and significantly maintained expressions of tight junction proteins and mucin protein and promotes recovery of the intestinal barrier. In addition, these supplementations regulate the composition of gut microbiota and improve the production of short-chain fatty acids (SCFAs), through enrichment of SCFA-producing bacteria, such as *Akkermansia* and *Ruminococcus* species. In summary, the synbiotic ameliorated the overall inflammatory status of the experimental UC model and showed a better treatment effect than *B. coagulans* or COSs did alone as revealed by the markers such as, colon length, IL-4 and Occludin levels.

**IMPORTANCE** Probiotic and prebiotic are believed to be useful in alleviating the inflammatory, thereby resolving or preventing the severity of UC. Spore-forming bacteria Bacillus coagulans show advantages of stability and probiotic effects, being suggested as the important probiotics for UC treatment. Here, we demonstrate that administration of *B. coagulans* spores, chitooligosaccharides (COSs), or the synbiotic attenuates DSS-induced colitis and significantly correlates with altered gut immune responses. The treatment effect of the synbiotic is inferred to be relied on the enrichment of probiotic bacteria, such as *Akkermansia* and *Ruminococcaceae* species, which are reported to be crucial important for gut health. Our findings facilitate the development of therapeutic and preventive strategies for UC using spore-forming lactic acid bacteria in combination with COSs.

Inflammatory bowel disease (IBD), including Crohn's disease (CD) and ulcerative colitis (UC), is characterized by the chronic inflammation of the gastrointestinal tract (1).The pathogenesis of IBD is complex and unclear, which may be caused by intestinal barrier dysfunction, immune system disorders and intestinal microbiota dysbiosis (2). In the past 40 years, the incidence of IBD has steadily increased in the world (3, 4). Western diet with rich energy and low nutrient density is considered a factor for the rising the incidences of IBD (3, 5). Drugs have beneficial effects; however, they also have adverse effects on IBD patients. On the other hand, dietary intervention is increasingly regarded as a preventive and corrective strategies to normalize dysfunctional microbiota, altered immunity, and barrier integrity functions (6). In this regard, probiotic and prebiotic are thought to be useful in alleviating the inflammatory, thereby resolving or preventing the severity of IBD (6–8). Probiotic and prebiotic can improve inflammatory parameters in the gut by modifying microbiota composition and metabolites, regulating secretion of immunomodulatory molecules, and protecting the colonic epithelial barrier (9–13). Synbiotics are the combination of probiotics and prebiotics, which interact positively and have potential preventive and therapeutic effects, and could play a synergistic role, bringing health benefits to the host (14, 15).

Recently, multiple evidences indicate that intestinal microbiota plays an important role in intestinal inflammation (16). The use of probiotics is a popular method to regulate intestinal microorganisms nowadays. Since the definition of probiotics, it has been confirmed that probiotics play roles in anti-inflammation, anti-oxidation, and regulation of intestinal microbes (17, 18). Bacillus coagulans is one of the probiotic species with increasing interests because this species forms stress-resistant spores that survive gastric transit, harsh manufacturing, and storage and delivery conditions (19, 20). It has been reported that the *B. coagulans* spores can be activated in the stomach through gastric acid to germinate and reach the intestinal tract for proliferation to playing beneficial function in the intestinal tract (21). Additionally, *B. coagulans* strains are known to have therapeutic effects because of their capacity to alter the host's microbiota, immunological system, and metabolism through the generation of antimicrobial substances, immune cell regulation, and other processes (22). Moreover, prebiotics, most of which are polysaccharides, are considered to be promising agents for improving colonic inflammation by regulating the intestinal microbiota and host metabolism to regulate intestinal barrier function and anti-inflammatory effects (23). Chitooligosaccharide (COS) is an oligomer of d-glucosamine, which is derived from decomposition or deacetylation of chitosan (24). In addition, many studies emphasize that COS has a positive impact on intestinal healthy, such as alleviating inflammation and the associated intestinal barrier damage in both dextran sulfate sodium (DSS)-induced colitis and acetic acid-induced colitis in the animal models (25, 26).

Although many previous studies have proved that *B. coagulans* has antioxidant activity, regulating intestinal microbiota and preventing inflammation (22), the mechanisms for UC treatment by *B. coagulans* are still covered. In this study, we found that *B. coagulans* FCYS01 can be grown on media with COSs as carbon source (Fig. S1), and oral administration of *B. coagulans* FCYS01 spores and COSs remarkably reduced the colitis severity, improved the immune function, restored the intestinal barrier, and promoted the steady state of gut microbiota of the DSS-treated mice, therefore, providing the evidence for beneficial effects of *B. coagulans* in combination of COSs on treatment of UC.

## RESULTS

### *B. coagulans*, COSs, and the synbiotic reduced DAI and macroscopic inflammatory markers in DSS-induced mice.

**FIG 1:**
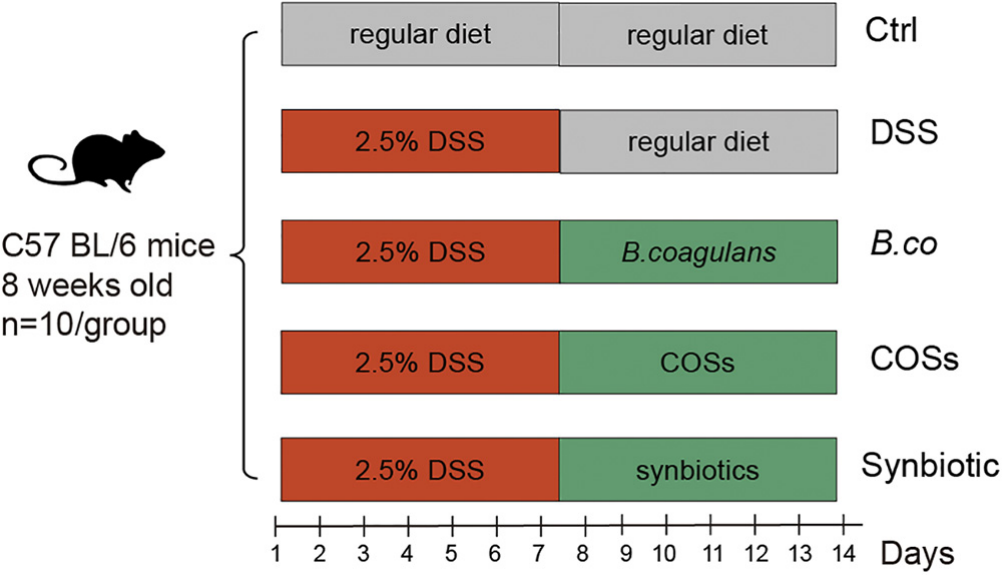
Animal experiment design of supplementation with *B. coagulans*, COSs, and synbiotic on DSS-induced colitis mice. Colitis was induced by administration of 2.5% DSS in drinking water for the first 7 days. *B. coagulans* spores (*B. co*), COSs, or the synbiotic (spores + COSs) were supplemented in the regular diet for the last 7 days. Ctrl: healthy control group fed with regular diet.

**FIG 2:**
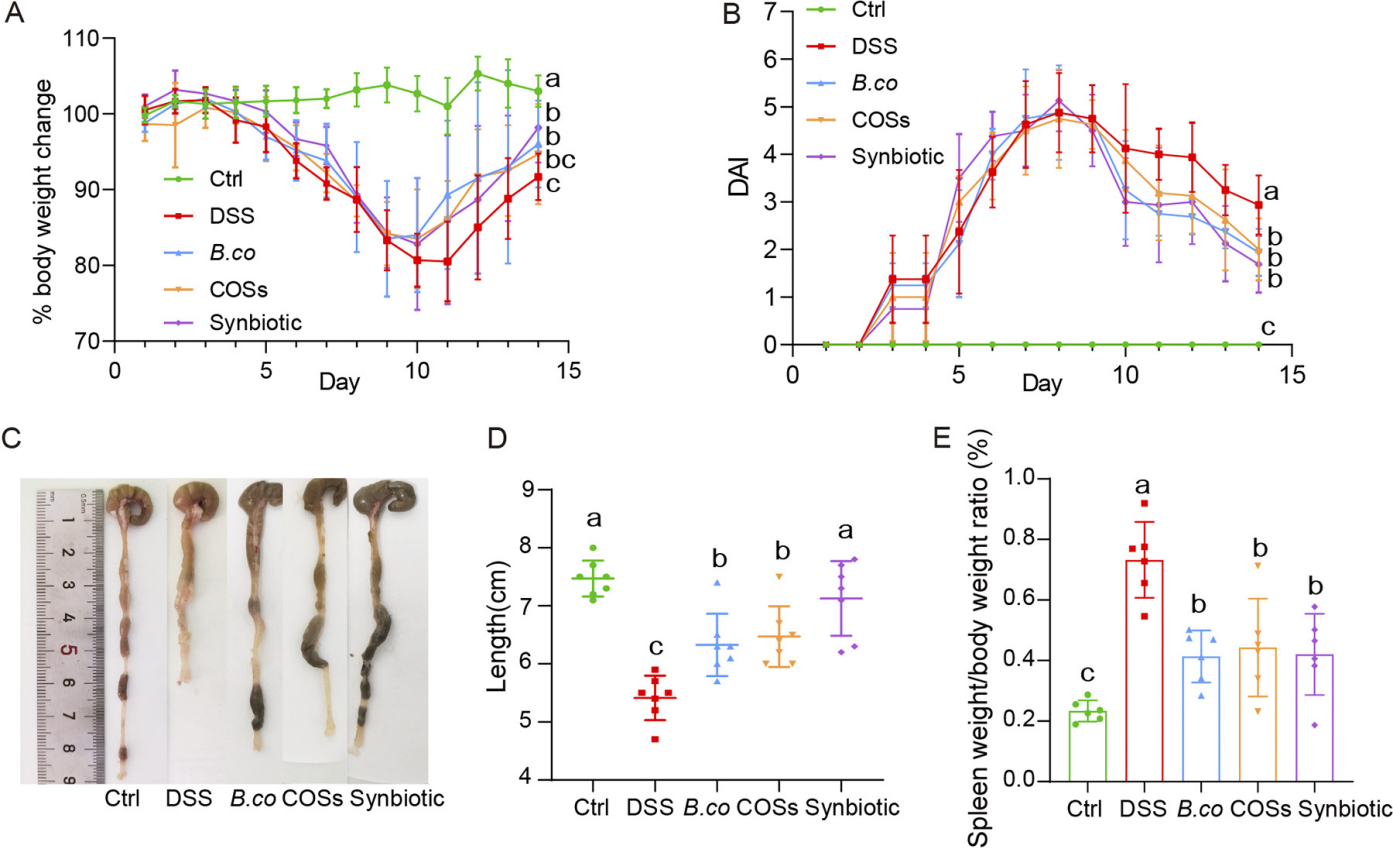
Treatment effect of *B. coagulans*, COSs, and synbiotic on DSS-induced colitis mice. (A) body weight change, (B) disease activity index (DAI), (C) macroscopic images of the colons, (D) colons length, (E) spleen weight/body weight ratio. Data are shown as means ± SEM (*n *= 10 per group). Data with different superscript letters (a, b and c) are significantly different (*P* < 0.05) according to two-way ANOVA (A and B) and one-way ANOVA (D and E) followed by Tukey’s test. Ctrl: healthy control; DSS: DSS-treated group; *B. co*, COSs, and Synbiotic: supplementation of *B. coagulans* spores, COSs, or spores + COSs, respectively.

To study the effect of *B. coagulans*, COSs and synbiotic (*B. coagulans* + COSs) on DSS-induced colitis in mice, we used 2.5% DSS in drink water for modeling for the first 7 days, followed by 7 days of daily oral administration of *B. coagulans* FCYS01 spores, COSs or synbiotic (Fig. 1). Administration of DSS resulted in significantly increase of colonic inflammation, as demonstrated by severe body weight loss and high DAI score (Fig. 2A and B). Compared with the DSS group, oral administration of *B. coagulans*, COSs and the synbiotic after 7 days promoted boosted recovery of body weight and reduced DAI values (Fig. 2A and B). At the end of the experiment on day 14, the average weight of DSS-treated mice in the synbiotic recovered best (98.17% of the first day), followed by *B. coagulans* group, COSs group and the DSS group (96.00%, 94.67% and 91.66% of the first day, respectively; Fig. 2A). Compared with the DSS group, the *B. coagulans* group and synbiotic group significant recovered the weight, while the COSs group has no significance (*P* = 0.0169, 0.0011 and 0.1796, respectively; Fig. 2A). The DAI values of all DSS-treated groups reached the highest points on day 8, and decreased afterwards (Fig. 2B). At the end of the experiment on day 14, DAI value was significantly higher for the DSS group (2.9 ± 0.6) compared to *B. coagulans*, COSs and the synbiotic groups (1.9 ± 0.5, 2.0 ± 0.6 and 1.7 ± 0.5; *P* = 0.0001, 0.0095 and 0.0003, respectively; Fig. 2B).

The evaluation of colon length affirmed the beneficial effects of *B. coagulans* and/or COSs in our study. The colons were significantly shorter after treatment with DSS, compared with colons from the control group (Fig. 2C and D). While oral administration of *B. coagulans* spores and/or COSs, the colons length extended (Fig. 2C and D). Although the colons from the *B.coagulans* and COSs groups were still shorter than that from the control group, they were also significantly longer than that of the DSS group (*P* = 0.001 and 0.0048, respectively; Fig. 2D). Importantly, there was no significant difference in colons length between the synbiotic group and the healthy control group (*P* = 0.816; Fig. 2D). In addition, the relative spleen weight of the mice in the DSS group were also significantly higher than that of *B. coagulans* group (*P* = 0.0004), COSs group (*P* = 0.0060) and the synbiotic group (*P* = 0.0019) (Fig. 2E). Together, these results indicated obvious treatment effect of the *B. coagulans* and the synbiotic on DSS-induced UC in mice.

### *B. coagulans*, COSs, and the synbiotic reduced histological damage of colon in DSS-treated mice.

**FIG 3:**
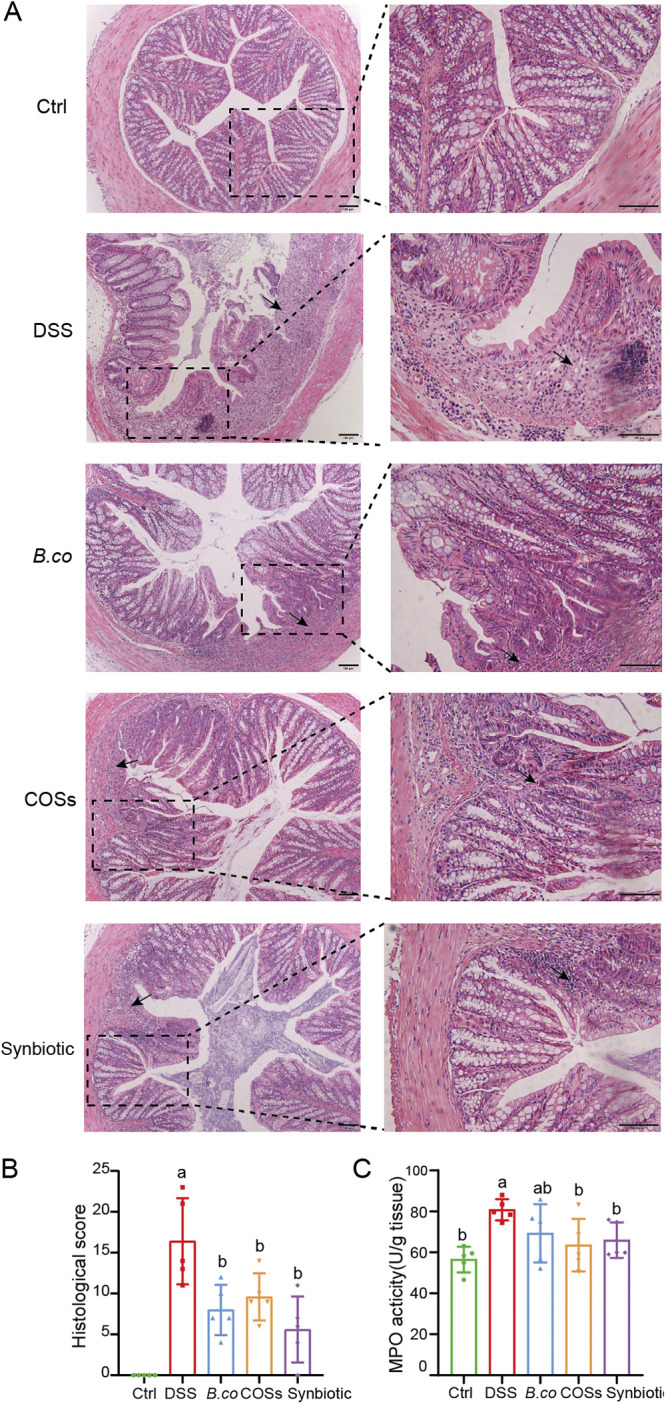
Treatment effect of *B. coagulans*, COSs, and synbiotic on DSS-induced colon injury and inflammation. (A) Microscopic images of proximal colonic tissues stained with H&E. Scale bars represent 100 μm. The right images are the enlarged part of the left images. Arrows indicate the damage of colon. (B) Histological score calculated after microscopic analyses of the colons. (C) Myeloperoxidase (MPO) activity in proximal colonic tissues. Data were shown as means ± SEM. Data with different superscript letters (a and b) were significantly different (*P* < 0.05) according to one-way ANOVA followed by Tukey’s test. Ctrl: healthy control; DSS: DSS-treated group; *B. co*, COSs, and Synbiotic: supplementation of *B. coagulans* spores, COSs, or spores + COSs, respectively.

H&E staining of colon tissue showed that in the control group, the colon epithelial tissue and the crypts were intact without infiltration of inflammatory cells (Fig. 3A). However, significant histological damage was found in the colons in the DDS group, including erosion or destruction of the intestinal epithelium, mucosal ulcer, crypt destruction, goblet cells depletion, and inflammatory cell infiltration (Fig. 3A). Compared with the DSS group, after oral administration of *B. coagulans* spores, COSs and the synbiotic, almost all of the colon tissue damage recovered, which evidenced by mostly intact intestinal epithelial structure, clearly visible crypts and goblet cells, and reduced inflammatory cells. (Fig. 3A).

To further evaluate the damage of the colon, we also performed histological scores on colon sections. The healthy control group showed no signs of histological colon damage (score 0), while, DSS resulted in a cumulative damage score of 16.4 ± 2.4 (Fig. 3B). Supplementation with *B. coagulans*, COSs and synbiotic induced promoted improved recovery of the damage, this resulted in a significant overall reduction of cumulative histological scores of colons (8.0 ± 1.3, 9 ± 1.3, 5.6 ± 1.8 for *B. coagulans*, COSs and the synbiotic, respectively). Analysis of myeloperoxidase activity showed that all three treatments successfully reduced neutrophil infiltration compared to the DSS group (Fig. 3C). COSs group and synbiotic group have a more significant effect in reducing MPO activity (*P* = 0.0236 and 0.0109, respectively), which was better than *B. coagulans* (*P* = 0.1279) compared with the DSS group (Fig. 3C).

### Improvement of immune regulation by *B. coagulans*, COSs, and the synbiotic in DSS-treated mice.

**FIG 4:**
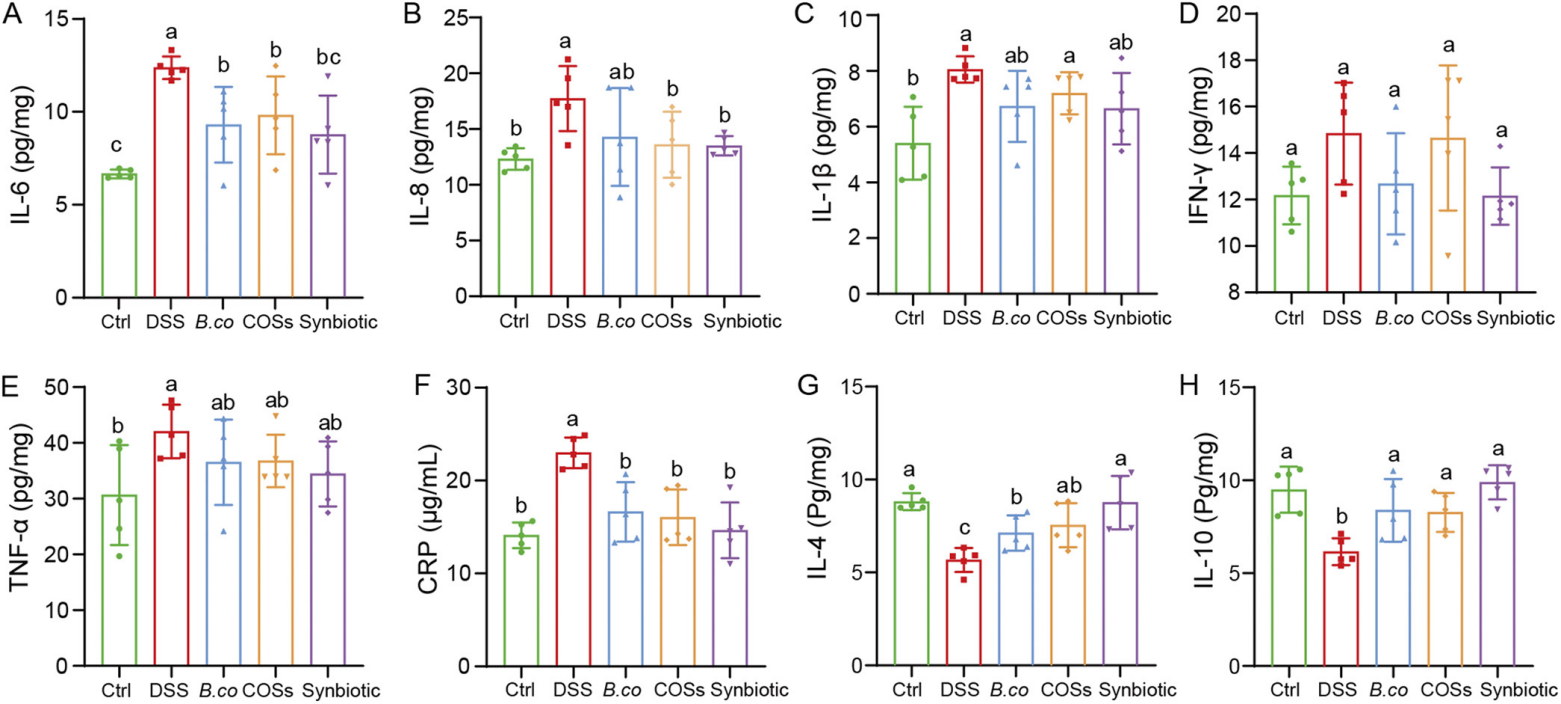
Effect of *B. coagulans*, COSs, and synbiotic on regulation of immune markers. Protein levels of cytokines, including (A) IL-6, (B) IL-8, (C) IL-1β, (D) IFN-γ, (E) TNF-α, (F) CRP, (G) IL-4, and IL-10 (H) of proximal colon tissues, were analyzed by ELISA. Data are shown as means ± SEM (*n *= 5 per group). Data with different superscript letters (a, b and c) are significantly different (*P* < 0.05) according to one-way ANOVA followed by Tukey’s test. Ctrl: healthy control; DSS: DSS-treated group; *B. co*, COSs and Synbiotic: supplementation of *B. coagulans* spores, COSs, or spores + COSs, respectively.

Proinflammatory factors and anti-inflammatory factors, which play important roles in the immune response in UC, were determined in this study. Overall, the concentration of the proinflammatory cytokines IL-6 (*P* = 0.0120, 0.0305 and 0.0062, respectively) and IL-8 (*P* = 0.1810, 0.0464 and 0.0142, respectively) in the colon were remarkably reduced in the *B. coagulans*, COSs, and synbiotic groups compared to the DSS group (Fig. 4A and B). However, the levels of proinflammatory cytokines, including IL-1β and IFN-γ and TNF-α in *B. coagulans*, COSs and synbiotic groups showed no significant difference with that in the DSS group (Fig. 4C to E). All three treatments also significantly reduced the concentration of serum CRP to the healthy control level, compared with that of the DSS group (Fig. 4F). Moreover, the levels of the anti-inflammatory cytokines IL-4 (*P* = 0.0075, 0.0071 and < 0.0001, respectively) and IL-10 (*P* = 0.0325, 0.0326 and 0.0003, respectively) in colon segments were significantly increased in the *B. coagulans*, COSs, and synbiotic treatment groups relative to the DSS group (Fig. 4G and H). In summary, the cytokines in the synbiotic group were more similar to the healthy control group than that in *B. coagulans* and COSs groups.

### *B. coagulans*, COSs and the synbiotic supplementation regulated the composition and SCFAs production of gut microbiota.

**FIG 5:**
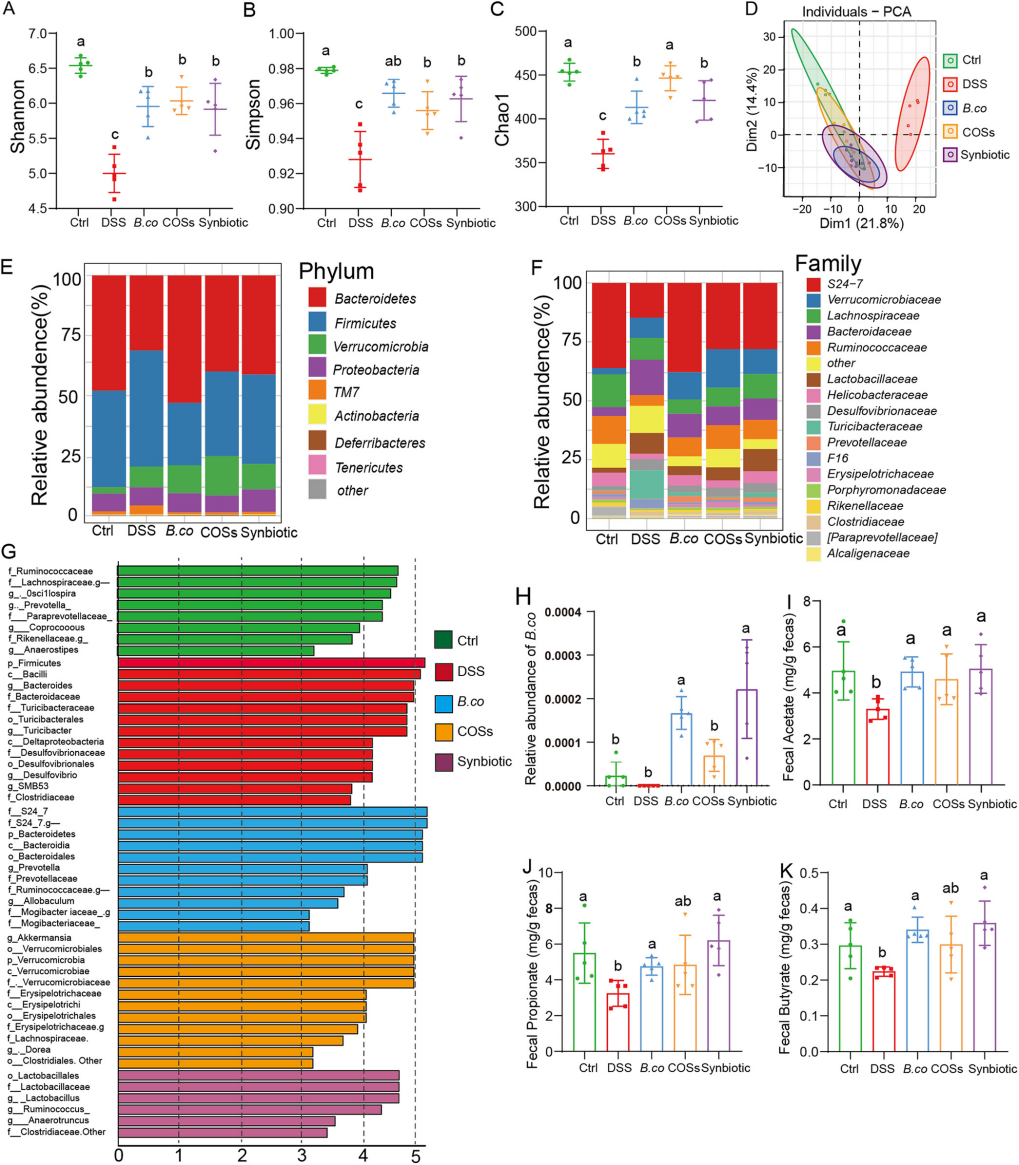
*B. coagulans*, COSs and synbiotic regulated the composition of gut microbiota. (A-C) Alpha-diversity indices. (D) Principal-component analysis of Bray-Curtis distance (the difference in PC1 and PC2 was 21.8% and 14.4%). (E and F) The relative abundance of fecal bacterial phylum (E), and family (F). (G) Analysis of differences in the microbial taxa by LEfSe in different groups. (H) The relative abundance of *B. coagulans*. Concentrations of fecal acetate (I), propionate (J), and butyrate (K). Ctrl: healthy control; DSS: DSS-treated group; *B. co*, COSs and Synbiotic: supplementation of *B. coagulans* spores, COSs or spores + COSs, respectively.

We further explored the impact of *B. coagulans*, COSs, and the synbiotic on the gut microbiota composition of the DSS-treated mice through 16S rRNA gene sequencing of fecal samples collected on day 14. Compared with healthy control group, the DSS administration significantly decreased the alpha-diversity of gut microbiota as indicated by Shannon, Simpson, and Chao1 (*P* < 0.0001, < 0.0001, < 0.0001, respectively) index (Fig. 5A to C). The alpha-diversity of gut microbiota was significantly increased in the groups supplemented with *B. coagulans*, COSs and the synbiotic as indicated by Shannon (*P* = 0.006, < 0.0001 and 0.0021, respectively), Simpson (*P* = 0.0015, 0.019, 0.0055, respectively) and Chao1 (*P* = 0.0014, 0.0001, 0.0012, respectively) index compared with the DSS-control group (Fig. 5A to C). Principal Components analysis (PCA) based on Bray-Curtis distance showed a separation in the gut microbiota structure among healthy control and DSS group (Fig. 5D). When *B. coagulans*, COSs, or the synbiotic were supplemented, the community clustering was significantly similar to that of the healthy group rather than the DSS group (Fig. 5D).

The difference of gut microbiota at phylum level showed that the microbiota structure was similar in each group, but the abundance of each component was different. The dominant bacterial community in each group were *Bacteroides*, *Firmicutes*, *Verrucomicrobia*, *Proteobacteria*, and *TM7*, *Actinobacteria*, *Deferribacteres*, and *Tenericutes* followed (Fig. 5E). Compared with healthy control group, the DSS administration significantly decreased the content of *Bacteroides*, *Proteobacteria*, in addition, the content of *Firmicutes*, *Verrucomicrobia*, and *TM7* are significantly increased. *Firmicutes* and *TM7* were remarkably decreased, and *Bacteroides*, *Verrucomicrobia*, and *Proteobacteria* were significantly increased following treatment with *B. coagulans*, COSs, or the synbiotic. While, *S24-7*, *verrucomicrobiaceae*, *Lachnospiraceae*, *Bacteroidaceae*, and *Ruminococcaceae* were predominant families in each group (Fig. 5F). Compared with healthy control group, the DSS administration significantly decreased the content of *S24-7*, *Lachnospiraceae* and *Ruminococcaceae*, significantly increased the content of *Verrucomicrobiaceae*, *Bacteroidaceae*, *Lactobacillaceae*, and *Turicibateraceae*. Moreover, relative to that of DSS group, following treatment with *B. coagulans*, COSs or the synbiotic, S24-7 was significantly increased, and the gut microbiota was restored to a composition similar to the noninflamed control group. Differentially abundant fecal bacterial taxa in DSS-treated mice in response to *B. coagulans*, COSs and synbiotic were further identified by LEfSe analysis (Fig. 5G). We found that four bacterial genera including *Bacteroides*, *Turicibacter*, *Desulfovibrio*, and *SMB53* were enriched in the DSS group, while the other three taxa were enriched including *S24-7*, *Allobaculum*, *Prevotella*, and *Ruminococcaceae* in *B. coagulans* group, *Akkermansia* in the COSs group, and *Lactobacillales*, *Lactobacillaceae*, *Ruminococcus*, and *Anaerotruncus* in the synbiotic group (Fig. 5G). These results indicated probiotic bacteria were enriched in the treatment groups, which might be one the mechanism to promote the health of DSS-treated mice in this study. In addition, further analysis the sequencing results of 16S rRNA showed that the relative abundance of *B. coagulans* in group *B. coagulans* and synbiotic increased significantly (Fig. 5H), which also provided evidence for the activation and germination of *B. coagulans* spores and their proliferation in the intestinal tract.

To further explore the effect of *B. coagulans*, COSs and synbiotic on the production of SCFAs, we measured the last day of the fecal concentrations of acetate, propionate, and butyrate. All three SFCAs were significantly reduced in the DSS group, while the supplementations promoted the production of SFCAs (Fig. 5I to K). Overall, the concentration of the SFCAs was remarkably elevated in the *B. coagulans*, COSs and their synbiotic treatments groups, including acetate (*P* = 0.0018, 0.0408 and 0.0093, respectively), propionate (*P* = 0.0047, 0.0603 and 0.003, respectively), and butyrate (*P* = 0.0001, 0.0680 and 0.0014, respectively) in fecal, compared with that of the DSS-induces group (Fig. 5I to K). These results suggest that synbiotic and *B. coagulans* were both associated with increased SFCAs in the gut compared to the control group.

### *B. coagulans*, COSs, and synbiotic restored the intestinal barrier in DSS-induced mice.

**FIG 6:**
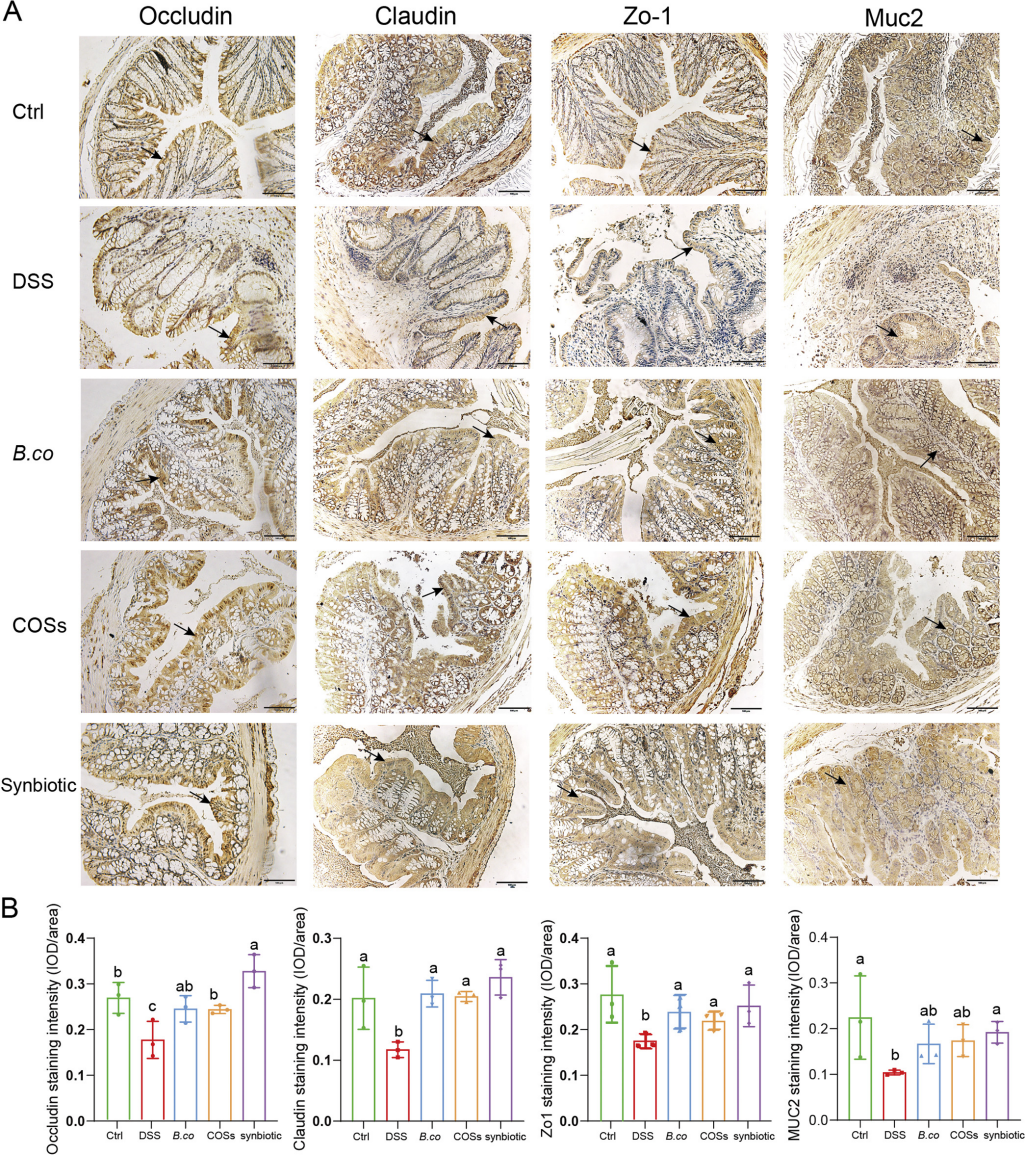
Effect of *B. coagulans*, COSs, and synbiotic on the expression of tight junction proteins. (A) Microscopic images of proximal colonic tissues stained with immunohistochemical of Occludin, Claudin, Zo-1, Muc2. Scale bars represent 100 μm. Arrows indicate the IHC positivity. (B) Mean optical densities with Image-pro plus soft of respective group is illustrated in the graph. Data are shown as means ± SEM. Data with different superscript letters are significantly different (*P* < 0.05) according to one-way ANOVA followed by Tukey’s test. Ctrl: healthy control; DSS: DSS-treated group; *B. co*, COSs, and Synbiotic: supplementation of *B. coagulans* spores, COSs, or spores + COSs, respectively.

To further verify the probiotic effect of *B. coagulans*, COSs and the synbiotic, the intestinal barrier integrity and colonic tight junction protein content were analyzed. The histology scores on these immunohistochemical sections are consistent with the results in Fig. 3B, the DSS group was the highest, and the score decreased after supplementation with *B. coagulans*, COSs and synbiotic (Fig. S2). The expression of tight junction proteins (Occludin, Claudin and Zo-1) were analyzed in the colonic tissue sections, overall, in DSS group, the mean optical density of the proteins was shallow, and the protein distribution was diffuse and discontinuous, indicating a low expression (Fig. 6A). Compared with control group, the level of TJ protein in the DSS group was significantly reduced, including Occludin (*P* = 0.0392), Claudin (*P* = 0.0497), and Zo-1 (*P* = 0.0471) (Fig. 6B). Moreover, the levels of the TJ protein expression were remarkably increased in the *B. coagulans*, COSs, and their synbiotic treatments group, including Occludin (*P* = 0.0670, 0.0421 and 0.0079, respectively), Claudin (*P* = 0.0006, 0.0033 and 0.0029, respectively), and Zo-1 (*P* = 0.0439, 0.0312 and 0.0478, respectively), except for Occludin (*P* = 0.0670) in group *B. coagulans*, compared with that of the DSS-induces group (Fig. 6B). The mean optical density of the TJ proteins in the synbiotic was the highest. In addition, we also performed immunohistochemical study on mucin protein Muc2, which mainly exists in and around goblet cells and is an important component of the mucus layer. Similar to TJ protein, the mucin Muc2 expression was the lowest in DSS group. After the treatments by *B. coagulans*, COSs and their synbiotic, the expression of Muc2 was remarkably increased (*P* = 0.0161, 0.0311 and 0.0006), compared with that of the DSS-induces group (Fig. 6B), which may be related to the recovery of intestinal barrier. In summary, administration of the synbiotic was most efficiency in recovery of the barrier integrity after DSS-treatment in mice.

## DISCUSSION

Dietary supplements of probiotics and prebiotics that function by regulating cytokines, colonic epithelial integrity, and gut microbiota are being widely investigated for the prevention or ameliorates of IBD (27, 28). The results of this study clearly indicated that the addition of *B. coagulans* spores, COSs or their synbiotic to the dietary strategy markedly reduced the symptoms and severity of DSS-induced colitis in mice. This was evidenced by the improvement of clinical symptoms, shortened colon, enlarged spleen, damaged colon tissue, inflammation disorders, intestinal microbiota disorders, reduced SCFAs levels and reduced tight junction protein levels in the DSS-induced model of colitis. Although we could not determine whether synbiotic supplementation directly stimulated the expression of TJs and Muc2 proteins, we observed that synbiotic significantly increased the expression of these proteins’ degradation by DSS, restoring them to that of the healthy group. These suggest that synbiotic supplementation significantly ameliorated the integrity of the epithelium. Taken together the results support a synergistic therapeutic effect between *B. coagulans* and COSs to restore epithelial integrity in DSS induced mice, thus supporting its application in UC. In addition, IBD includes both Crohn's disease and ulcerative colitis (UC) (29), although the use of probiotic strains is usually invalid in Crohn's disease (30, 31), probiotic intervention can improve mild to moderate ulcerative colitis (32). The result in our manuscript is that *B. coagulans* and COSs intervention can attenuate colitis induced by DSS, which is consistent with previous reports (26, 33, 34). Our results laid the research foundation for that *B. coagulans* and COSs can treat UC.

Due to the advantages of higher acid resistance and better stability compared to other probiotics (35), many Bacillus spp., such as *B. coagulans*, B. Subtilis, *B. pulmilus*, *Bacillus* OJ, B. licheniformis, and *B. fusiformis* are currently used as probiotic dietary supplements (36). Bacillus coagulans can regulate the immune system, promote the recovery of intestinal microbes to normal, and has antiviral activity. Because of its reported abilities to modulate the endogenous gut microbiota, bind and inhibit pathogenic bacteria, restore intestinal epithelial barrier function, and regulate host immunity, *B. coagulans* may be useful for treating intestinal diseases. (22, 37). The IBD triggers aberrant inflammatory responses resulting in increased accumulation of proinflammatory mediators and thus further exacerbating the inflammation cascade and tissue damage (38). *B. coagulans* could regulate the expression level of IL-10 under inflammatory conditions (33, 34), which indicates that the anti-inflammatory effect of synbiotic may be related to the main immunomodulatory ability of *B. coagulans* in this study, thus supporting its application in the treatment of UC. Impaired epithelial barrier caused by disruption of the intestinal epithelial TJs is a significant marker in clinical IBD patients (39), resulting in intracellular spaces between adjoining epithelial cells and the movement of harmful substances through the intracellular spaces (40). *B.coagulans* could upregulate the expression of TJs and mucin protein in colitic mice (33, 34), which is consistent with the observation of supplementing *B.coagulans* in our study. The structure, composition, and status of the gut microbiota are closely related to the host health. Metabolites of the microbiota, such as SCFAs, may play an anti-inflammatory role, regulate the intestinal immune activity, and enhance epithelial barrier function (41–43). Previous study (34) has confirmed that *B. coagulans* could upregulate the levels of SCFAs in colitic mice, which is consistent with the observation of supplementing *B.coagulans* in our study. The significantly increased levels of SCFAs after the treatments of *B.coagulans* may also be associated with the positive effects of colon histology, barrier integrity, and reduced severity of disease in DSS induced mice.

Although the association between *B. coagulans* and the colitis has been widely concerned and verified, it has been reported that *B. coagulans* was added at the beginning of the experiment to ameliorates the colitis induced by DSS in a preventive way (34). In this experiment, DSS was first used to induce colitis in mice, and then *B. coagulans*, COSs or their synbiotic was supplemented to treat the colitis. These three supplementations significantly improved the colitis induced by DSS. In addition, gut microbiota of mice treated with *B. coagulans* has not been reported in previous reports (34). In our study, through 16S rRNA gene sequencing, we revealed that the supplemented of *B. coagulans*, COSs or their synbiotic significantly increased the community richness and diversity, and probiotic bacteria that have been shown to produce SCFAs were enriched (43). *Ruminococcaceae*, *Akkermansia*, and *Ruminococcus* enriched in the *B. coagulans*, COSs, and synbiotic group, respectively, which have been proved to be related to the production of SCFAs and have strong probiotic properties (44). Therefore, the synbiotic may directly or indirectly affect the ability of the probiotic intestinal microbiota to produce SCFAs, which is beneficial in regulating UC inflammation (45). Interestingly, the *Lactobacillus* (46) exerts probiotic effects by regulating the balance of intestinal microbiota and inhibiting the growth of potentially pathogenic bacteria was also enriched in the synbiotic group. The therapeutic effect on colitis of COSs could be due to intestinal barrier protection by regulating the production of intestinal epithelial tight junction protein and intestinal mucus (26). In addition, the relative abundances of *Turicibacter* and *Desulfovibrio* were enriched in the DSS group and were significantly decreased after *B. coagulans*, COSs, or synbiotic treatment. Some *Turicibacter* bacteria are reported often positively correlated with host inflammation (47) and was abundantly detected in tumor-bearing mice (48). Identification of *Desulfovibrio* in an immunodeficient patient with advanced UC has been reported, suggesting that the organism may be an opportunistic human pathogen (49). In summary, our results demonstrate that *B. coagulans*, especially in combination with the COSs prebiotic, may represent a novel treatment option for UC.

## MATERIALS AND METHODS

### Species and oligosaccharides.

The probiotic species *B. coagulans* FCYS01 was isolated from fresh fecal of cattle. The spores were produced through incubation of *B. coagulans* FCYS01 in basic sporulation medium and collected by centrifugation. The COSs (5 to 10 oligomers of d-glucosamine) were enzymatically prepared from commercial chitosan using the chitosanase produced from a Pichia pastoris recombinant strain (24).

### Animals.

Fifty C57BL/6 (8-weeks old) mice of both sexes with average weight of 25 g were purchased and carried out in the Center for Experimental Animals, Huazhong Agricultural University, and housed in 25°C with a 12-h day/night light cycle. Individual body weights were assessed daily including over an initial acclimation period of 7 days. All mice had *ad libitum* access to radiation-sterilized rodent feed pellets and autoclaved tap water for drinking during experiments. All experimental animals have been approved by the Animal Ethics Committee of the Animal Experimental Ethical Inspection of Laboratory Animal Centre, Huazhong Agriculture University (Ethics Approval Number: HZAUMO-2021-0103).

### Study design and treatments.

Following 1 week of acclimation, mice were randomly allocated into the following five groups (10 mice per group, 5 mice per cage): (1) Healthy control group, (2) DSS-treated group, (3) DSS-treated mice supplemented with *B. coagulans* FCYS01 spores (*B.co* group), (4) DSS-treated mice supplemented with COSs (COSs group), and (5) DSS-treated mice supplemented with *B.co* and COSs (synbiotic group). The experiment lasted 14 days for all the mice, in addition, 2.5% DSS in the drink water was used to induce colitis for the first 7 days and *B. coagulans* spores, COSs or synbiotic (spores + COSs) was supplemented in the regular diet for the last 7 days (except the healthy group). The *B.co* group received probiotic *B. coagulans* FCYS01 spores of 10^9^ CFU/day/mouse by oral administration. The COSs group received COSs of 80 mg/day/mouse by oral administration. The synbiotic group received *B. coagulans* FCYS01 spores of 10^9^ CFU/day/mouse and COSs of 80 mg/day/mouse by oral administration. At the 15th day, all mice were sacrificed by anesthesia with chloral hydrate. Blood, spleen, colon, kidney, liver, intestine, and cecum were collected from all mice. After the experiments, all mice were burned.

### Clinical scoring and histological analysis.

A Disease Activity Index (DAI) was determined daily in all mice by scoring for body weight change, stool consistency and blood in feces during the experimental period. Scoring standards refer to previous experiments (33). Briefly, DAI was scored as follows: (a) weight loss (0% = score 0, 1 to 5% = score 1, 6 to 10% = score 2, 11 to 15% = score 3); (b) Consistency of stools (normal = 0, soft but still formed = 1, very soft/loose stools = 2, diarrhea/watery stools = 3); (c) blood in stool (negative hemoccult = 0, positive hemoccult = 1, blood traces in stool visible = 2, rectal bleeding = 3). Fecal samples from mice were collected at the end of the experiment (day 14) and stored at −80°C for SCFAs and microbiota analyses.

After sacrificing the mice, the colons were excised from the cecum to the anus. Spleen weight and colon length were recorded for the measurement of macroscopic markers of inflammation. For histological analysis proximal colon samples (*n* = 4 per group) were formalin-fixed and sectioned for hematoxylin-eosin (H&E) staining, slides stained with H&E were graded blindly for the severity of tissue damage at regions based on the previously described scoring system (33). Briefly, the frequency of inflammation distribution scored 0 to 3, crypt distortion and ulceration scored 0 to 5, tissue damage scored 0 to 3, inflammatory infiltration scored 0 to 3, goblet cell loss scored 0 to 3, and mucosal thickening (edema) scored 0 to 3. All sections were scanned by Nikon Eclipse 80i microscope (Nikon, Kobe, Japan).

### Tissue cytokine measurements and serum C-reactive protein analysis.

The proximal colon tissue samples were homogenized after adding PBS (pH 7.4) at 4°C. Supernatant was carefully collected after centrifuging for 20 min at 12, 000 g. Aliquots of the supernatant were used for ELISA. The cytokine levels in colon tissue (*n* = 5 per group) were determined by immunoassay using Mouse ELISA kit following the manufacturer’s instructions. The levels of C-reactive protein (CRP) in serum (*n* = 5 samples/group) were analyzed using Mouse C-Reactive Protein/CRP Quantikine ELISA kit following the manufacturer’s instructions. All kits were purchased from Mei Mian Biotechnology Co., Ltd., Jiangsu, China.

### Immunohistochemical detection of tight junction proteins.

Immunohistochemical detection of epithelial tight junction proteins, including Occludin, Claudin, Muc2, Zo-1 was performed using a Rabbit specific HRP Detection IHC kit (Cat: SA00001-2, Proteintech, China) following the manufacturer’s instructions and as previously described (34). Anti-Occludin (Cat: 27260-1-AP), anti-Claudin (13050-1-AP, Proteintech, China), anti-Muc2 (Cat: 27675-1-AP), anti-Zo-1 (Cat: 21773-1-AP) antibodies were used for incubating with the proximal colonic sections overnight at 4°C. Computer-assisted image analysis was performed with a Nikon Eclipse 80i microscope (Nikon, Kobe, Japan), and Image Pro Plus 7.0 software (Media Cybernetics, Inc., Rockville, MD, USA). The expression of tight junction proteins was blindly assessed by choosing random per five number of crypts of each sample to assess mean intensity (*n* = 3 per group).

### Microbiota analysis through 16S rRNA high-throughput sequencing.

The V3-V4 region of the 16S rRNA genes was PCR amplified from a DNA aliquot of the extracted fecal samples (*n* = 5 per group), and the PCR products were sequenced by Illumina sequencing at Novogene (Tianjin, China). The double-ended sequences in FASTQ format were screened for quality one by one using sliding window method. The high-quality sequences obtained above were dechimeric and subjected to merging and OTU partitioning with 97% sequence similarity using Usearch software (http://www.drive5.com/usearch/), and the sequence with the highest abundance in each OTU was selected as the representative sequence of the OTU. The taxonomy of each 16S rRNA gene sequence was analyzed against the SILVA (SSU132) 16S rRNA database. Alpha diversity and beta diversity were analyzed by QIIME (50). Linear discriminant analysis (LDA) effect size (LEfSe) differences among biological groups were tested for significance using non-parametric factorial Kruskal-Wallis test and Wilcoxon rank-sum test (51).

### Quantification of SCFAs.

SCFAs were extracted and analyzed from each fecal samples, with some modifications as described previously (52). SCFAs, including acetate, propionate, and butyrate in fecal samples were quantified with gas chromatography. Briefly, 50 mg of fecal samples were weighed, dissolved, homogenized, and then centrifuged at 3, 000 × *g* for 5 min. The supernatant was adjusts pH to 2 to 3 with HCl, and then centrifuged at 12, 000 × *g* for 10 min, the last filtered through a 0.22 μm sterile membrane, kept in a 2 mL screw-cap vial, and then subjected for SCFAs analysis with an gas chromatography system (Agilent Technologies, USA).

### Statistical analysis.

All statistical analyses were performed using GraphPad Prism 8 and SPSS. One-way and two-way analysis of variance (ANOVA) was used to evaluate the data, and then Tukey's back testing was performed to determine the statistical difference between each group.

### Date availability.

The data sets supporting the conclusions of this article are available in the NCBI Sequence Read Archive (SRA) repository under accession number PRJNA801837.
