# Supplementary material for: Bacillus coagulans in Combination with Chitooligosaccharides Regulates Gut Microbiota and Ameliorates the DSS-Induced Colitis in Mice
Source: Microbiol Spectr. 2022 Jul 28;10(4):e00641-22. doi: 10.1128/spectrum.00641-22 (PMC9430726; doi:10.1128/spectrum.00641-22)
Supplement: Supplemental file 1 — Supplemental material. Download spectrum.00641-22-s0001.pdf, PDF file, 0.1 MB [file spectrum.00641-22-s0001.pdf]

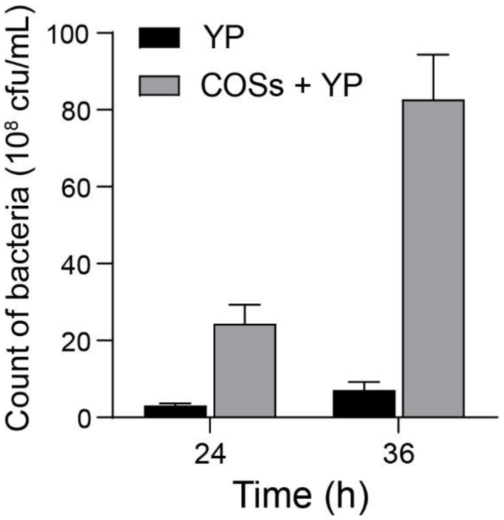

Supplementary Figure 1. The count of bacteria of *B. coagulans* FCYS01 with COSs as carbon source. YP: Yeast extract 10 g /L and Tryptone 20 g /L; COSs +YP: COSs 10 g /L, Yeast extract 10 g /L and Tryptone 20 g /L.

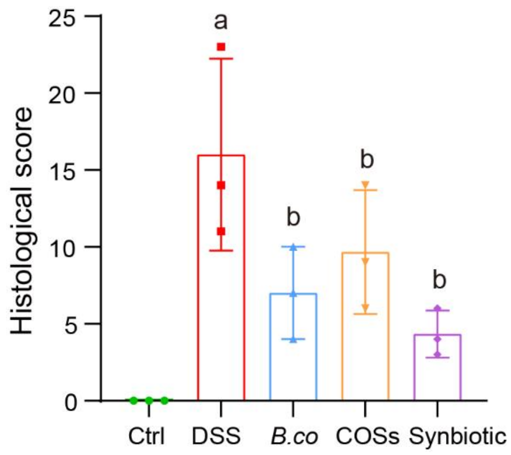

Supplementary Figure 2. Histological score calculated after microscopic analyses of the colons used for immunohistochemical analysis. Data were shown as means ± SEM. Data with different superscript letters (a and b) were significantly different ($p < 0.05$) according to one-way ANOVA followed by Tukey's test. Ctrl: healthy control; DSS: DSS-treated group; *B. co*, COSs and Synbiotic: supplementation of *B. coagulans* spores, COSs or spores + COSs, respectively.
